# Canine distemper virus infection of vaccinal origin in a 14-week-old puppy

**DOI:** 10.1177/10406387241229436

**Published:** 2024-02-16

**Authors:** Emily Rätsep, Davor Ojkic

**Affiliations:** Animal Health Laboratory, Kemptville, University of Guelph, Guelph, Ontario, Canada; Guelph, University of Guelph, Guelph, Ontario, Canada

**Keywords:** canine distemper virus, encephalitis, modified-live virus vaccine, Rockborn strain of canine distemper virus, vaccination

## Abstract

The body of a 14-wk-old puppy (*Canis familiaris*) was submitted to the Animal Health Laboratory, University of Guelph, Ontario for postmortem examination following a history of intermittent anorexia and lethargy progressing to pyrexia, pruritic skin rash, mucoid nasal discharge, decreased mentation, dysphagia, muscle twitches, and focal seizures. Gross examination revealed rhinitis and pulmonary edema. Histologically, there was fibrinonecrotizing bronchopneumonia, tracheitis, and neutrophilic and lymphohistiocytic rhinitis; rarely within the cortical gray and white matter of the brain were small clusters of glial cells, with rare individual neutrophils in the choroid plexus. Although canine distemper was suspected, none of the usual supportive histologic lesions of distinct syncytial cells, viral inclusion bodies, or demyelinating leukoencephalitis were observed. Lung and brain tissues were PCR-positive for canine distemper virus (CDV), and CDV was detected immunohistochemically in the brain. The agent from the PCR-positive sample from the brain was genotyped and was a 99.9% match to the CDV Rockborn strain, indicating that the disease agent in our case was vaccinal in origin. Our unusual case highlights the possibility of reversion to virulence in a modified-live virus vaccine, and the occurrence of a disease in the absence of a full complement of the usual and compatible histologic lesions.

Canine distemper virus (CDV; *Paramyxoviridae*, *Morbillivirus canis*) can cause subacute-to-acute, systemic, and/or neurologic disease in dogs and other carnivorous species.^5,10,14,17–19^ With the increased use of a modified-live virus (MLV) vaccine against CDV, clinical disease in domestic animals has become increasingly rare. However, although uncommon, CDV infection can still cause disease in vaccinated animals. Emergence of new wild-type strains resulting in inadequate vaccine protection, complications by lingering maternal immunity, improper vaccine handling or vaccination protocols, vaccination failure in an immunocompromised host, and reversion to virulence of a MLV vaccine have been reported.^1–3,5–9,12,15–17,19,20^ Although rev-ersion to virulence is considered a potential cause of disease in vaccinated animals and has been reported for other viral diseases^4,13^ or in other species,^5,14^ there are few definitive reported cases in the literature of CDV MLV vaccine reversion in canids.^1^ We report here a case of a 14-wk-old puppy with rhinitis, gliosis, and bronchopneumonia consistent with vaccination-associated canine distemper.

A 14-wk-old puppy was presented for postmortem examination at the Animal Health Laboratory (University of Guelph, Ontario, Canada) following a history of intermittent anorexia and lethargy since 10 wk of age. Various potential causes including intussusception, intestinal foreign body, hypothyroidism, hepatic shunt, and cardiac disease were ruled out with no change in presentation. Initial vaccines (canine morbillivirus, adenovirus, parainfluenza virus, and parvovirus) were given at 7 wk, but the 12-wk booster was delayed because the puppy was too sick for revaccination. The puppy had not yet been vaccinated for rabies. Clinical findings were progressively reported as pyrexia and lethargy with pruritic skin rash, green mucoid nasal discharge, decreased mentation, dysphagia, muscle twitches, and ventral strabismus of the right eye within 48 h of euthanasia. Focal seizures (4 reported in 1 d) were the ultimate reason for electing euthanasia.

On postmortem examination, purulent catarrhal rhinitis and pulmonary edema were noted. Routinely collected, representative tissue samples were fixed in 10% neutral-buffered formalin and processed routinely for the production of H&E-stained sections. Histologically, changes in the lungs were florid, with marked alveolar necrosis, fibrin deposition, and abundant neutrophils (Fig. 1A). Neutrophilic and lymphohistiocytic rhinitis was confirmed; rarely, in sections taken at the level of the mamillary bodies and intermediate hypothalamus within the cortical gray and white matter of the brain, were small clusters of increased parenchymal glial cells (Fig. 1B). These glial cells were confirmed by immunohistochemistry for ionized calcium-binding adaptor molecule 1 (Iba1; anti-Iba1 polyclonal antibody 019-19741, Wako Chemicals), detected through an alkaline phosphatase polymer detection system (UltraView alkaline phosphatase; Roche); FastRed (Roche) was used as a substrate chromogen and Harris hematoxylin as a counterstain (Fig. 1B, inset). Rare individual mature neutrophils were occasionally seen in the choroid plexus. Other histologic findings included lymphohistiocytic tracheitis, presumed secondary to the pulmonary changes, and localized mild hepatic portal bridging fibrosis with occasional mild biliary hyperplasia (etiology undetermined but not considered to have been extensive enough to have had functional significance). The combination of the initial gross and histologic findings (glial nodules, fibrinonecrotizing bronchopneumonia, and choroid plexus neutrophils) suggested that acute septicemia and possible aspiration pneumonia were potential causes of the clinical decline of the dog. Although bronchopneumonia was present, no distinct syncytial cells or viral inclusions were observed in any tissue nor were any histologic changes supportive of demyelinating leukoencephalitis observed. Other findings typically associated with canine distemper such as enamel hypoplasia, hyperkeratosis of the footpads, and conjunctivitis were also not observed.^17^

**Figure 1.**
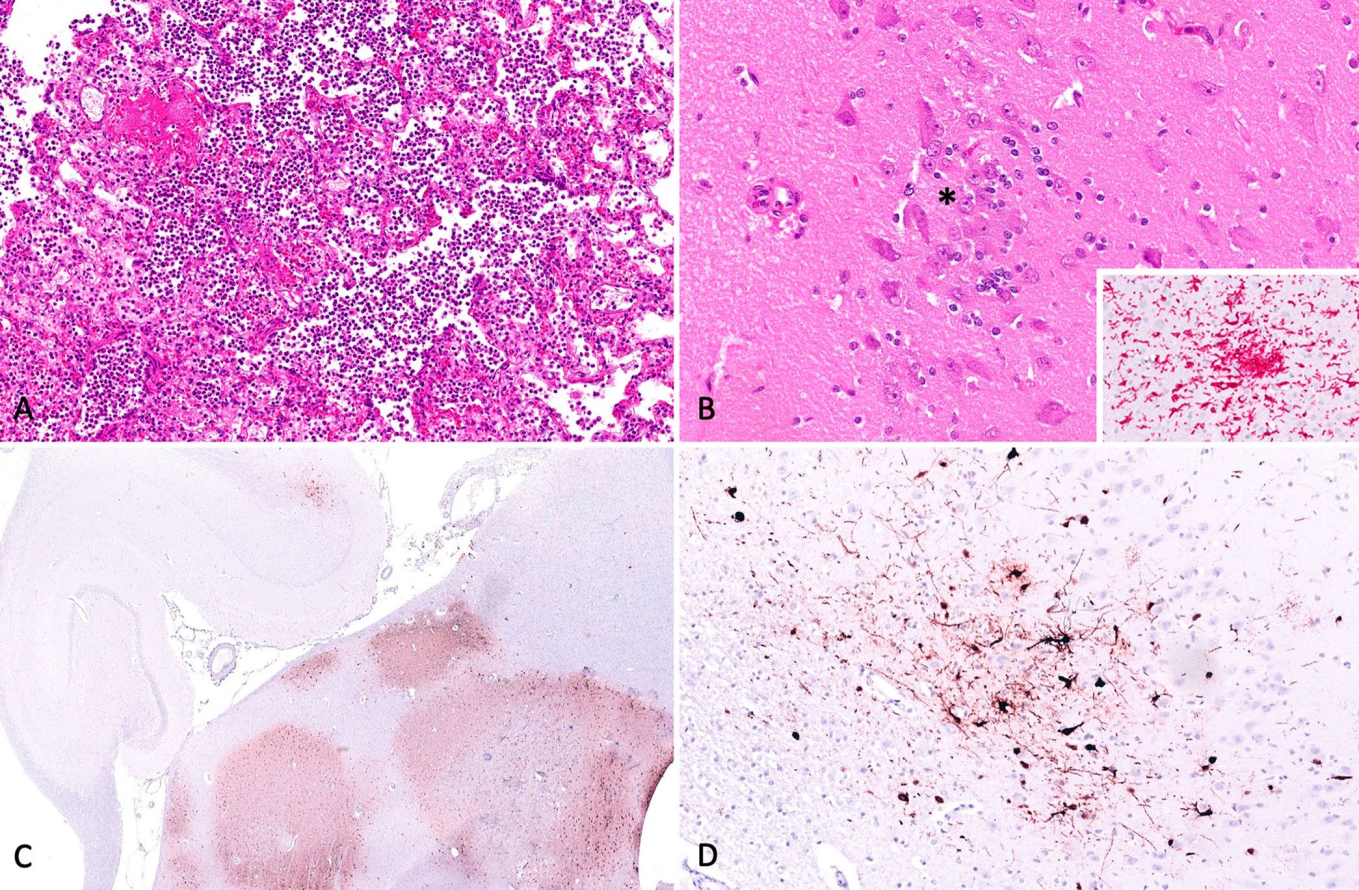
Representative microscopic features of the lung and brain lesions in a 14-wk-old puppy with a canine distemper virus (CDV) infection of vaccinal origin. **A.** Necrosis of alveolar walls in the lung, with fibrin deposition and numerous degenerate neutrophils and macrophages. H&E. **B.** Rare clusters of glial cells (asterisk) in the cortical gray matter. H&E. Inset: intense immunostaining of glial cells in brain for Iba1. Immunohistochemistry (IHC). **C.** Hippocampus with immunoreactivity for CDV antigen. IHC. **D.** Higher power view of brain with clusters of neuronal cell bodies and nerve processes with intense immunoreactivity for the CDV antigen.

Considering the clinical history and reported change in mentation, testing for CDV by PCR^6^ was carried out on lung and brain tissue, and CDV antigen was identified in both with cycle thresholds of 22.1 and 25.3, respectively. Immunohistochemistry (anti-CDV monoclonal antibody CDV-NP-0.5; VMRD) was performed on brain for further confirmation, and CDV was detected through a goat anti-rabbit horseradish peroxidase–labeled polymer detection system (EnVision+ system; Dako); NovaRed (Vector) was used as a substrate chromogen and Harris hematoxylin as a counterstain (Fig. 1C, 1D). Sections of lung from a known CDV-infected raccoon were used as positive tissue (run) controls. For negative reagent controls, duplicate sections of each control and test tissue were subjected to the same immunohistochemical procedure with substitution of antibody diluent for the primary antibody. Lung and brain were submitted for routine aerobic culture and plated on Columbia blood and McConkey agar plates. After overnight incubation at 35°C for 24 and 48 h, the bacteria grown were identified (MALDI-TOF mass spectrometer; Bruker). *Staphylococcus pseudintermedius* was identified in both brain and lung tissue, suggesting that a concurrent bacterial process was present, possibly secondary to aspiration and systemic spread, and could explain some of the more neutrophilic changes observed in the lung and brain.

The agent in the PCR-positive sample from the brain was genotyped and was a 99.9% match to the CDV Rockborn strain (Table 1), attenuated in canine kidney cells and used since the 1960s in several live-attenuated CDV vaccines. This strain has a potential for reversion to virulence and has been sporadically implicated in post-vaccinal disease cases.^1,16^

**Table 1.** Percentages of identities comparing hemagglutinin sequence of the clinical sample (brain from the 14-wk-old puppy) with canine distemper virus (CDV) vaccine gene sequences from GenBank.

CDV vaccine (GenBank accession)	Brain
Rockborn-Candur (GU266280)	99.9%
VanguardPlus/Pfizer (FJ461702)	99.9%
NobivacDHPPI/Intervet (FJ461701)	92.5%
Galaxy/SP/FortDodge (FJ461708)	92.5%
Intervet/NobivacPuppyDP (FJ461709)	92.5%
Lederle (EF418782)	92.5%
Onderstepoort/Convac (Z35493)	92.5%
SnyderHill/ATCC (JN896987)	92.4%
Virbac/CanigenDHPPI (FJ461710)	92.3%
Onderstepoort (EU143737)	91.9%

Vaccine-associated canine distemper cases have been reported in various breeds following vaccination with attenuated-live CDV vaccines.^1–3,7,8,15,16,20^ Clinical signs typically developed within 3 wk of vaccination and could include seizures and circling or changes in behavior. Most cases describing CDV in vaccinated animals were determined to be due to emergence of a new wild-type strain or were suspected to be the result of an inadequate immune response to a vaccine or vaccine failure.^8,15,20^ Reports of reversion to virulence of the CDV vaccine are rare,^7^ and most were demonstrated under experimental conditions (serial passage in dogs).^1,12^ Reversion to virulence is most commonly reported in immunosuppressed animals (immunocompromised or animals with concurrent infections, e.g., parvovirus), or is demonstrated in isolated cases as post-vaccinal encephalitis.^1,4–7,11,12,16,17^ In these and more acute cases, clinical signs associated with CDV, such as oculonasal discharge, diarrhea, pneumonia, and CNS disturbances, have been observed. Although histologically, in most reported cases, typical lesions associated with CDV infection are observed in cortical and central gray matter; only in one paper, in African hunting dogs, did lesions lack CDV-specific intranuclear or intracytoplasmic inclusion bodies and also lack prominent demyelination.^5^ To our knowledge, our case is the second report of canine distemper following reversion to virulence of a CDV vaccine in which neither inclusion bodies nor demyelination were observed, and the first report in a domestic canid, given that, in a search of Google, PubMed, CAB Direct, Web of Science, and Scopus using search terms “Canine distemper vaccine reversion to virulence,” we retrieved no cases describing reversion to virulence of a CDV vaccine in a domestic canid that lacked a description of inclusion bodies or typical lesions described in association with CDV infection. In our case, demonstration of CDV antigen in neurons confirmed the diagnosis.^5,7^

In our case, not only was CDV antigen expressed in neural tissue and detected by PCR assay in the lung and the brain, but CDV hemagglutinin gene sequencing allowed confirmation of the virus material being of vaccine strain origin. Our case serves as a reminder of the possibility, although uncommon, of reversion to virulence in a MLV vaccine.
